# Childhood trauma, midbrain activation and psychotic symptoms in borderline personality disorder

**DOI:** 10.1038/tp.2015.53

**Published:** 2015-05-05

**Authors:** K Nicol, M Pope, L Romaniuk, J Hall

**Affiliations:** 1 Division of Psychiatry, University of Edinburgh, Kennedy Tower, Royal Edinburgh Hospital, Edinburgh, UK; 2 Neuroscience and Mental Health Research Institute, Cardiff University, Cardiff, UK; 3 MRC Centre for Neuropsychiatric Genetics and Genomics, Cardiff University, Cardiff, UK

## Abstract

Childhood trauma is believed to contribute to the development of borderline personality disorder (BPD), however the mechanism by which childhood trauma increases risk for specific symptoms of the disorder is not well understood. Here, we explore the relationship between childhood trauma, brain activation in response to emotional stimuli and psychotic symptoms in BPD. Twenty individuals with a diagnosis of BPD and 16 healthy controls were recruited to undergo a functional MRI scan, during which they viewed images of faces expressing the emotion of fear. Participants also completed the childhood trauma questionnaire (CTQ) and a structured clinical interview. Between-group differences in brain activation to fearful faces were limited to decreased activation in the BPD group in the right cuneus. However, within the BPD group, there was a significant positive correlation between physical abuse scores on the CTQ and BOLD signal in the midbrain, pulvinar and medial frontal gyrus to fearful (versus neutral) faces. In addition there was a significant correlation between midbrain activation and reported psychotic symptoms in the BPD group (*P*<0.05). These results show that physical abuse in childhood is, in individuals with BPD, associated with significantly increased activation of a network of brain regions including the midbrain in response to emotional stimuli. Sustained differences in the response of the midbrain to emotional stimuli in individuals with BPD who suffered childhood physical abuse may underlie the vulnerability of these patients to developing psychotic symptoms.

## Introduction

Borderline personality disorder (BPD) is a common psychiatric condition, affecting ~1–3% of the general population, and is the most common personality disorder.^1^ Individuals with a diagnosis of BPD experience a range of symptoms, including affective lability and transient psychotic experiences. This combination of symptoms led to the initial characterization of the condition as being on the ‘borderline' between psychosis and neurosis, giving rise to its name.^2^ The aetiology of BPD is complex and both genetic and environmental factors are recognised as contributing to risk for the disorder.^3, 4^ Childhood adversity is a widely accepted risk factor for BPD, with the majority of sufferers disclosing physical or sexual abuse or emotional neglect in childhood.^5, 6^

Several studies have shown that individuals with BPD have altered responses to emotional stimuli, including subtle deficits in recognising negative facial emotions such as disgust and anger.^7, 8^ There is also increasing evidence that neutral or positive facial expressions are more commonly perceived as negative by those with BPD.^9, 10, 11^ Furthermore, previous work has revealed a correlation between facial emotion recognition in the disorder and experience of childhood physical abuse,^12^ suggesting a potential link between childhood adversity and later responses to emotional stimuli in BPD.

Emotional stress is known to increase the prevalence and experience of psychotic symptoms in BPD,^13, 14^ illustrating the sensitivity of sufferers to emotionally arousing stimuli. Research has indicated that childhood adversity may contribute to risk of developing psychotic symptoms more generally,^15, 16^ therefore childhood trauma may contribute to vulnerability for the development of psychotic symptoms in BPD. The mechanism associating childhood adversity and the development of psychotic symptoms in disorders such as BPD is, however, unclear. One possibility is that early-life experience modifies the function of the midbrain dopaminergic system. Dopamine signalling dysfunction has been established as playing an important role in the symptoms of psychosis experienced in schizophrenia^17^ and its receptors are the common target of antipsychotic therapeutics, which have been shown to have some efficacy in BPD, although effect sizes are small potentially reflecting selective efficacy for subclasses of symptoms in BPD, such as transient psychotic symptoms.^18, 19, 20^

Here, we sought to investigate brain activation in response to emotional stimuli in BPD. On the basis of previous work, we hypothesised that brain activation in response to emotional faces in BPD would be modified by prior experience of childhood adversity and would relate to current symptom severity, in particular, severity of psychotic symptoms.

## Materials and methods

### Participants

Twenty individuals with a diagnosis of BPD were recruited from outpatient populations. DSM-IV (Diagnostic and Statistical Manual of Mental Disorders, fourth edition) criteria of BPD was established using the SCID-II (Structured Clinical Interview for DSM Disorders-II). The BPD group consisted of 17 females and 3 males, mean age 35.6 (range 20–53 years) and mean intelligence quotient (IQ) 114.8 (assessed by the National Adult Reading Test). Of these, 12 were being treated with antipsychotic medication, and 15 with antidepressant medication. Sixteen healthy control participants were recruited from community volunteers, comprising 14 females and 2 males, mean age 35.7 (range 21–51 years) and mean IQ 113.3. Controls were also assessed for BPD using the SCID-II. Exclusion criteria for all participants included a history of bipolar I disorder or schizophrenia, current alcohol or drug dependency, or any neurological illness. In addition, exclusion criteria for healthy control participants included a diagnosis of BPD or other personality disorder. Comorbid psychiatric diagnoses were established using the SCID-I interview (in both BPD participants and controls) and case note review. In both control and BPD participants, psychotic symptoms (over the previous 2 weeks) were rated using the positive symptom component of the Positive and Negative Symptoms Scale (PANSS),^21^ current symptoms of depression were rated using the Hamilton Rating Scale for Depression,^22^ and current symptoms of mania were rated using the Young Mania Rating Scale.^23^ Severity of BPD symptoms was assessed using the Zanarini Rating Scale for BPD.^24^ Participants also completed the childhood trauma questionnaire (CTQ), a self-report measure of incidence and severity of childhood trauma.

The study was approved by the Lothian Research Ethics Committee. All participants gave written informed consent following a period of at least 24 h consideration with access to the study information sheet. All participants had the opportunity to discuss the study, and understood that they were free to withdraw at any time. Participants who withdrew from the study were not disadvantaged in any way.

### fMRI task

In the scanner, participants performed a task that involved viewing photographs of faces on a computer screen, and selecting whether they thought the face was male or female, indicated by pressing the corresponding response button. To ensure that participants had a full understanding of the task, screen-shots of the images used were shown to each person before entering the scanner and a complete explanation of the task was provided. Faces were chosen from the Ekman series of emotional faces^25^ and displayed either a fearful or a neutral expression. The task was made up of six blocks, with each containing six images of faces. Within each block, all of the faces displayed the same emotion—either neutral or fearful—with half being female and half male, shown in a random order. Pictures of the same individuals were shown in both the fear and neutral blocks, differing only in the emotion portrayed. The blocks alternated between fear and neutral and were interspersed with rest blocks lasting 12.5 s during which participants were asked to fixate on a cross in the centre of the screen. Task blocks commenced with a 1 s visual prompt (‘Gender?') followed by each face being shown on the screen for 3.5 s with a 0.5 s inter-stimulus interval between faces. A response (male or female) had to be made within the 4-s presentation to be registered, providing a measure of compliance with the task. Two versions of the task were used, one showing neutral faces in the first block, and the other showing fearful faces in the first block. The version of the task (neutral block first or fear block first) shown to each participant was randomised.

### fMRI data acquisition and analysis

Imaging data was acquired using a 3T Siemens Magnetom Verio Syngo MR scanner. Functional imaging scans were acquired using the following parameters: repetition time 1560 ms, echo time 26 ms, flip angle 66°, 26 slices (slice thickness 5 mm), field of view 220 × 220 mm, voxel size 3.5 × 3.5 × 5 mm. A high resolution T1 MPRAGE structural image was also acquired with repetition time 2300 ms, echo time 2.98 ms, flip angle 90′, field of view 256 × 256 ms, in-plane resolution 256 × 256, 160 interleaved slices, providing a final resolution of 1 × 1 × 1 mm.

Data processing was carried out using Statistical Parametric Mapping Software (SPM8, Wellcome Trust Centre for Neuroimaging) and based on MATLAB software version 7.13 (MathWorks). The first six images were discarded, and the remaining images realigned to the mean image. The realigned images were then co-registered to the corresponding T1-weighted anatomical images, and these were spatially normalised into the Montreal Neurological Institute brain template. Finally, the normalised data was smoothed with a three-dimensional isotropic Gaussian kernel (8 mm full width at half maximum).

Statistical analysis was performed in SPM8. At the individual participant level, the data were modelled with two conditions (‘fear' and ‘neutral') each modelled as a boxcar convolved with a canonical haemodynamic response function. Parameters representing the participants movement during the scan were also entered into the model as covariates of no interest. Contrast images were generated for each participant for the contrasts of interest. The primary comparison of interest was the contrast of fearful versus neutral faces. Second-level analysis was carried out to compare differences within and between groups using *t*-tests. Within-group regression analysis was performed across all participants in the BPD group using scores on the PANSS, Hamilton Rating Scale for Depression, Young Mania Rating Scale and CTQ, to identify areas of brain activation that correlated with clinical data. All statistical maps were thresholded at a level of *P*<0.005 uncorrected and regions were considered significant at *P*<0.05 at the cluster level, corrected for multiple comparisons. In line with our prior hypotheses that childhood trauma would have a particular impact on brain regions involved in emotional and motivation processing, we applied a small volume correction for activation within the amygdala. A midbrain/ventral striatum small volume correction was also applied as used previously,^26^ supported by previous results indicating a relationship between childhood trauma and brainstem responses to fearful faces in women.^27^

Further statistical analysis was carried out using SPSS software version 19 (IBM, Armonk, NY, USA). Demographic and clinical characteristics between groups were compared, and activation data was extracted from SPM for use in SPSS correlation analysis in the BPD group. Causal mediation analysis was carried out using R version 3.0.2 to investigate the link between childhood trauma, midbrain activation and psychotic symptoms.

## Results

### Demographic, clinical and behavioural data

There was no difference in age (*t*_1,36_=3.82, *P*=0.97; range 20–53 in controls and 21–51 in the BPD group) or IQ (*t*_1,31_=0.56, *P*=0.58) between groups. Five control participants did not complete the National Adult Reading Test and these individuals were excluded from IQ analysis. The BPD group scored significantly higher on the Hamilton Rating Scale for Depression (*t*_1.36_=8.07, *P*<0.001), Young Mania Rating Scale (*t*_1,36_=2.92, *P*<0.01), PANSS (*t*_1,36_=−4.34, *P*<0.001) and CTQ (*t*_1,35_=6.99, *P*<0.001) than the control group. All participants showed good engagement with the fMRI task (99.4% overall response rate). There was no difference between participants with BPD and control participants in terms of their reaction times or accuracy for gender judgements for neutral faces (*P*>0.1 for both). However, participants with BPD were marginally slower than controls when making gender judgements on fearful faces (1.54 s versus 1.38 s; *t*_1,35_=2.27, *P*<0.05) and were less accurate in making these judgements (93% accuracy versus 99% accuracy, *t*_1,35_=2.21, *P*<0.05). Participant information is detailed in Table 1.

### Brain activation to emotional faces

**Figure 1:**
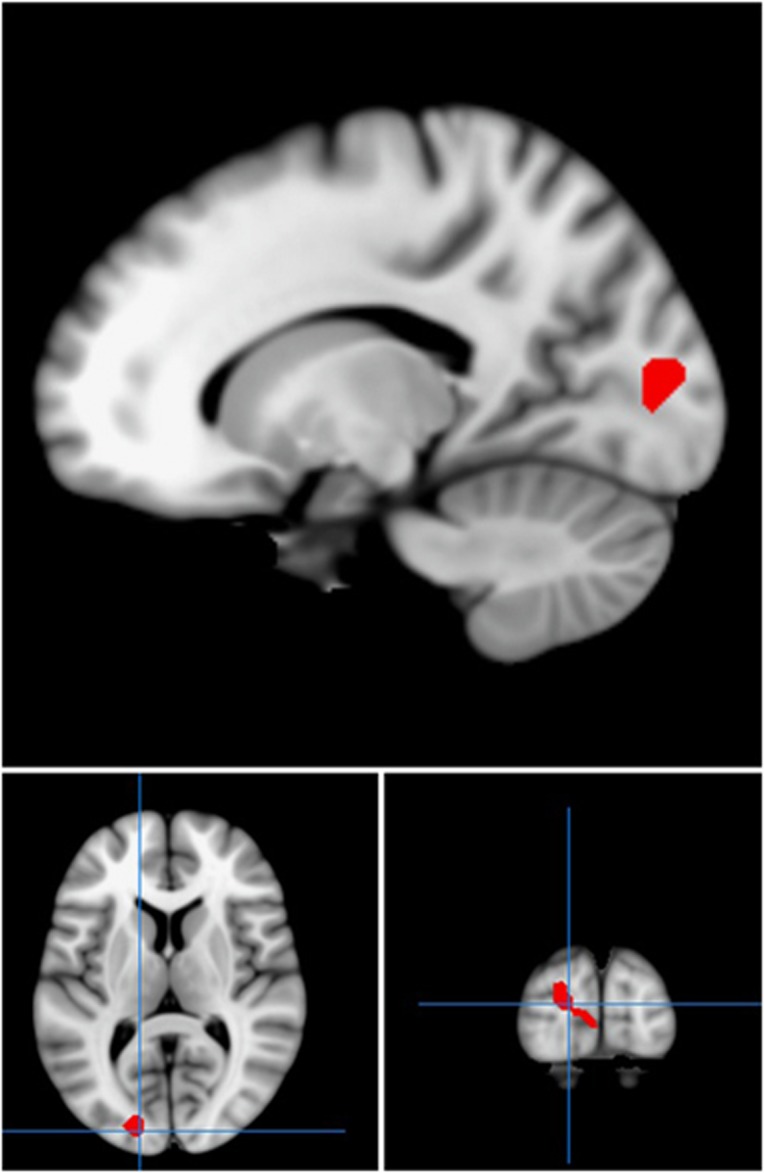
Regions of statistically greater activation in the right cuneus in the control group compared with the borderline personality disorder group, in response to fearful versus neutral faces. Red areas show activation differences meeting threshold *P*<0.005 across whole brain, superimposed on the mean T1 image.

Across all participants, there were significant bilateral increases in activation when viewing fearful compared with neutral faces in the fusiform gyrus bilaterally (*P*<0.001, *K*_E_=401, *Z*=4.21, co-ordinates 30, −82, −2 and *P*=0.001, *K*_E_=240, *Z*=5.34, co-ordinates −27, −82, −2) and amygdala bilaterally (within amygdala small volume correction, *P*=0.04, *K*_E_=7, *Z*=3.25, co-ordinates 18, 4, −20 and *P*=0.02, *K*_E_=17, *Z*=3.54, co-ordinates −24, −10, −14). There was also significant activation in the medial frontal gyrus (*P*=0.003, *K*_E_=192, *Z*=4.41, co-ordinates 42, 11, 25). Comparing activation between groups, significantly greater BOLD responses were noted in control participants than in those with BPD in the right cuneus (*P*=0.03, *K*_E_=238, *Z*=3.72, co-ordinates −18, −88, 7) to fearful versus neutral faces (Figure 1).

### Effect of childhood experience

**Figure 2:**
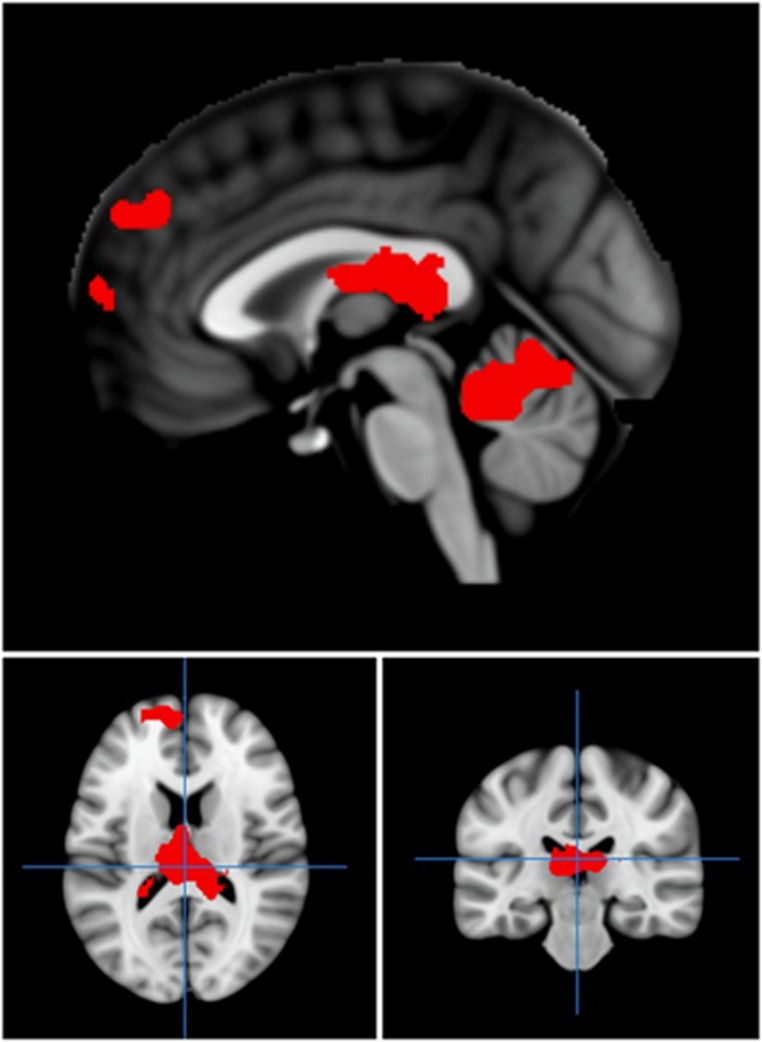
Regions of activation in medial frontal gyrus and cerebellum which correlated significantly with childhood physical abuse, as assessed by the CTQ, in those with borderline personality disorder. Red areas show activation meeting threshold *P*<0.005 across whole brain, superimposed on the mean T1 image. CTQ, childhood trauma questionnaire.

**Figure 3:**
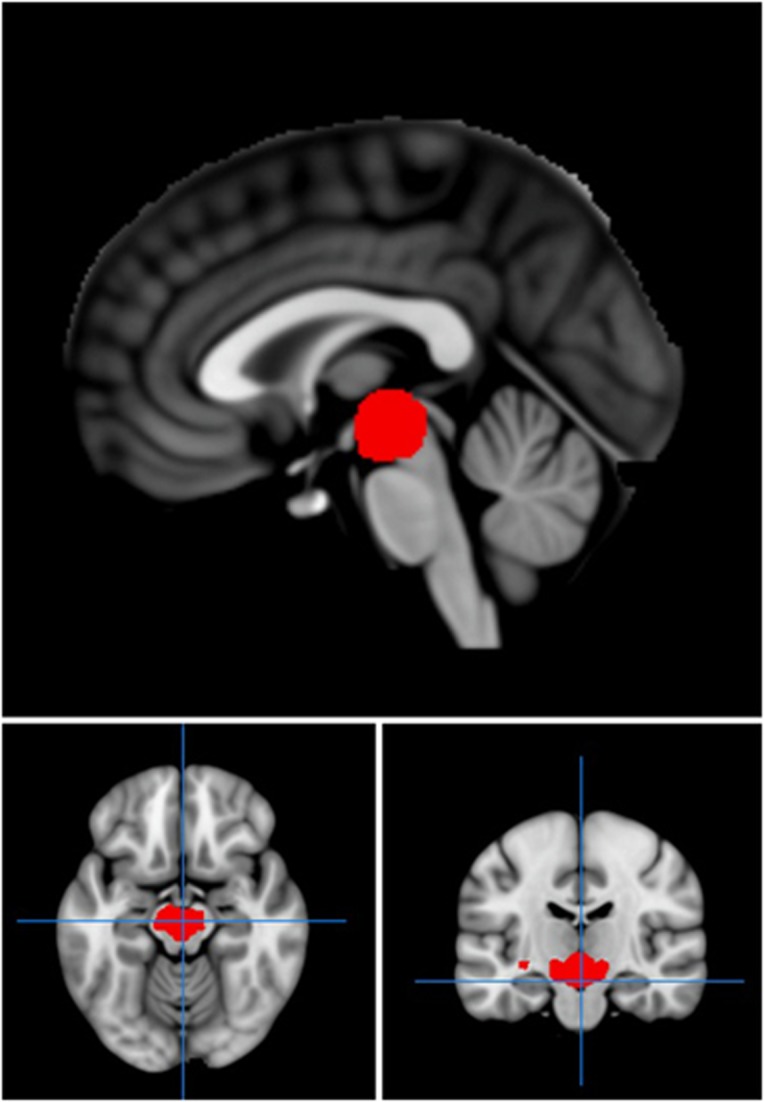
Region of activation in midbrain, which correlated significantly with childhood physical abuse, as assessed by the CTQ, in those with borderline personality disorder. Red areas show activation meeting threshold *P*<0.005 within a midbrain/ventral striatum mask, superimposed on the mean T1 image. CTQ, childhood trauma questionnaire.

We next investigated the relationship between experience of childhood trauma, as measured by the CTQ, and brain activation to emotional faces in those with a diagnosis of BPD. We specifically focussed on childhood experiences of physical abuse and emotional abuse based on previous behavioural data showing that these areas of childhood experience specifically had an effect on later responses to emotional faces, a finding that was not replicated with other subscales of the CTQ.^12^ Regression of scores for prior physical abuse taken from the CTQ against brain activation to fearful (versus neutral) faces revealed a network of brain activation related to increased physical abuse scores in BPD. Increased activation as a function of severity of prior physical abuse was found in the medial frontal gyrus (*P*=0.04, *K*_E_=240, *Z*=3.82, co-ordinates −24, 44, −2), pulvinar (*P*=0.007, *K*_E_=348, *Z*=3.93, co-ordinates −3, −25, 13) and cerebellum (*P*=0.03, *K*_E_=246, *Z*=3.66, co-ordinates 6, −52, −17; Figure 2). Following application of a midbrain/ventral striatum mask,^26^ significant activation was also noted in the midbrain (*P*=0.04, *K*_E_=82, *Z*=3.66, co-ordinates −6, −16, −14; Figure 3). The relationship between physical abuse and midbrain activation remained significant after controlling for other CTQ subscales. There were no significant correlations of brain activation with emotional abuse scores or total CTQ scores, supporting the specificity of the findings to physical abuse.

### Brain activation and correlation with psychotic symptoms

We sought to determine whether increased midbrain activation in participants with BPD who had suffered physical abuse in childhood was related to current psychotic symptoms. Analysis of extracted data for the peak voxel of activation within the midbrain within the BPD group showed a significant correlation with reported positive psychotic symptoms as assessed by the PANSS (*P*<0.05, *r*=0.45, *n*=20), with a particularly strong association seen between midbrain activation and the PANSS item for persecutory beliefs (P6). No association was seen between peak activation in the pulvinar, medial frontal gyrus or cerebellum and psychotic symptoms.

We finally investigated whether midbrain activation mediated a relationship between childhood physical abuse and psychotic symptoms in adulthood in our BPD sample. Mediation analysis revealed a strong trend towards significance (*P*=0.06), indicating that childhood physical abuse may increase later psychotic symptoms through alterations in midbrain activity.

### Effects of medication status

We finally determined whether any of the above effects could be accounted for by medication effects within the BPD group. Analysis of extracted data demonstrated that there were no diffrerences between BPD participants treated with antidepressants or antipsychotics and those not treated with such medications in terms of brain activation in any of the key areas of activation including the cuneus and midbrain.

## Discussion

We have investigated brain responses to fearful facial stimuli in individuals with BPD and their relationship to self-reported experiences of childhood trauma and psychotic symptoms. Group differences in brain activation between participants suffering from the disorder and controls viewing emotional faces were restricted to the cuneus. However, within the BPD group, a strong relationship was observed between childhood experiences of physical abuse and greater activation of the midbrain, medial frontal gyrus, pulvinar and cerebellum to emotional stimuli. Midbrain activation to emotional faces was additionally correlated with severity of current psychotic symptoms, especially persecutory beliefs. These results support an association between childhood trauma and long-lasting changes in brain responses to emotional stimuli. Although it is not possible to ascertain from the current study whether this effect is specific to BPD, such changes may contribute to the development of psychotic symptoms in adulthood, particularly at times of negative emotional stimulation.

Direct comparison of brain activation in participants with BPD compared with controls viewing fearful faces revealed group differences in the activity of the cuneus. This difference may be attributed to the role of the cuneus in visual processing, potentially reflecting altered responsiveness of individuals with BPD to visual cues such as those presented, which could be perceived as negative or threatening. Previous studies involving BPD populations have also reported decreased activation^28^ and reduced functional connectivity^29^ of the cuneus in this disorder. The cuneus is also believed to have a role in theory of mind—the ability to attribute mental states to others—and studies involving healthy populations have revealed increased activity in this region during tasks involving theory of mind.^30, 31^ Taken together with our results, these findings suggest that cuneus dysfunction may contribute to deficient emotional processing in BPD.

Previous studies have identified a relationship between childhood trauma and altered emotion regulation, as well as behavioural responses to emotional stimuli in BPD.^12, 32^ Childhood adversity has also been strongly associated with the development of symptoms of the disorder, and more generally with the development of psychotic symptoms across a range of disorders.^5, 6, 15^ We investigated the relationship between childhood adversity and brain responses to emotional stimuli in participants with BPD. Using this analysis, we identified a significant association between brain activation in a network of regions including the midbrain, medial frontal gyrus, pulvinar and cerebellum, and previous experience of physical abuse as measured by the CTQ.

The midbrain is the key site of dopaminergic afferents to the limbic system and is known to be involved in emotion processing.^33^ Dysfunction of this brain region is widely theorised to contribute to the development of psychotic symptoms in schizophrenia.^17, 34^ The present results suggest that, in patients with BPD, childhood physical abuse results in an increased response of the midbrain to negative (fearful) emotional stimuli. We also found that individuals with BPD who experienced childhood physical abuse may be particularly vulnerable to the development of psychotic symptoms, via midbrain dysfunction, when exposed to negative emotional stimuli in adulthood. These findings provide a potential biological rationale for the use of antipsychotic medications to ameliorate psychotic symptoms in BPD.^35^ However, fMRI is only an indirect measure of dopamine system activation and the results would be strengthened by future studies using more direct measures such as positron emission tomography. Furthermore, the psychotic symptoms reported by BPD sufferers were generally at the milder end of the range of severity as assessed by the PANSS, and it may be these symptoms such as suspiciousness and paranoid ideation, to which BPD sufferers are particularly vulnerable.

The present study identified additional areas of increased BOLD activation in response to fear versus neutral faces that correlated significantly with physical abuse in the BPD group. Greater activation was observed in the pulvinar, which is believed to be responsible for relaying fear information to the amygdala.^36^ A study with healthy volunteers found that the pulvinar was activated specifically in response to fearful faces,^37^ highlighting its importance in fear responses. The increased pulvinar activation observed in the current study may indicate a heightened fear response in individuals with BPD who suffered physical abuse in childhood. This is in keeping with previous work, which found that those with this diagnosis tended to judge neutral social cues as more threatening compared with controls.^12^ The correlation of activation of the cerebellum to emotional stimuli with physical abuse is likely to reflect the increasingly recognised role of the cerebellum in processing affective and social information, and, in particular, in fearful emotion processing.^38, 39^ The frontal gyrus has been implicated as being involved in facial emotion processing, particularly in differentiating between neutral faces and those showing emotion,^40^ and increased activation in this area may reflect heightened sensitivity to emotional compared with neutral facial expressions.

The specificity of the current findings to physical abuse warrants further comment. It is possible that physical abuse has a particularly strong relationship with subsequent brain responses to fear-related stimuli in adulthood, resulting in the activation of limbic/motivational ‘salience' networks. However, an alternative possibility is that physical abuse is most clearly recalled by participants, or has greater discriminatory power due to the range of responses reported. However as other behavioural changes, such as social judgement decision biases, are more strongly correlated with sexual abuse in BPD,^12^ we suggest that physical abuse may have a particular impact on fear processing in adulthood.

The present study demonstrates a significant association between childhood trauma and brain activation in BPD, and suggests a link between childhood physical abuse and psychotic symptoms in adulthood, which may be mediated through altered midbrain activation. To the authors' knowledge, this is the first time such correlations have been identified in a BPD population. These results may help explain the sensitivity of many individuals with the disorder to negative emotional stimuli, and, in particular, the tendency of sufferers to develop psychotic symptoms at times of emotional stress. Altered midbrain activation may also underlie the response to antipsychotic treatment seen in patients with BPD. The results also support the use of early psychological interventions in individuals with this diagnosis to ameliorate negative responses to environmental stimuli, and highlight the importance of managing emotional stressors in sufferers. They illustrate the key role of early-life experience in modulating midbrain activation, which may be of relevance to a range of psychiatric disorders, particularly those in which psychotic symptoms are a significant feature.

There are some limitations to the study, which should be acknowledged. First, the majority of participants in the BPD group were taking medication at the time of the study. It is possible, though unlikely, that medication effects may have contributed to the group difference in activation noted in the cuneus and in the correlations noted between brain activation and childhood trauma. However, the likelihood of this is very low, given the variation of medication use across participants and the specific effects of childhood abuse seen in the within-group correlation analyses. Second, the BPD participants in the current study were predominantly female consistent with clinical populations in the UK but not with epidemiological studies, which point to a more balanced sex ratio.^3^ Participants in the current study also had a relatively broad age range, but this was closely balanced across groups. Third, the participants in the current study were of higher-than-average IQ, potentially representing the local demographics and a tendency for more able individuals to take part. Fourth, the findings within the midbrain were within an *a priori* area of interest, although these findings were strengthened by the subsequent specific association of activation in this region with severity of psychotic symptoms. Fifth, there was insufficient range of CTQ scores in the control population to determine whether the relationship between physical abuse and midbrain activation is also seen in individuals without BPD, which would require larger samples. Sixth, although the current research has identified a trend towards midbrain activation mediating the relationship between childhood physical abuse and psychotic symptoms in adulthood in BPD, this finding fell just short of formal statistical significance. Further investigation is required, ideally using larger sample sizes, to better determine the role of the midbrain in the development of psychotic symptoms. Similarly, this relationship in other psychiatric disorders should be investigated to elucidate whether this effect is specific to BPD.

Nonetheless, the present findings highlight the damaging effects of adverse childhood experiences into adulthood, and support early intervention for those at risk of developing BPD. Early intervention strategies, for example, engagement with families or carers, family intervention and tailored psychotherapy may prove effective. Our results also provide some support for the use of drugs targeting the dopamine system in BPD, but more research is needed.^41^ Overall, the study emphasises the importance of continuing biological research into BPD to further our understanding of the condition and to identify suitable therapeutic or alternative therapies for those suffering from the disorder.

## Figures and Tables

**Table 1:** Participant information

*Demographics*	*BPD*			*Healthy control*			P
	n	*mean*	*s.d.*	n	*mean*	*s.d.*	
Age	20	35.8	8.6	16	34.8	9.6	0.97
IQ	20	114.8	7.9	16	114.5	6	0.58
CTQ (total)	20	52.9	19.8	16	13.1	11.5	<0.001
Emotional abuse		11.4	3.3		3.9	1.9	<0.001
Physical abuse		12.5	7.5		5.5	1.3	<0.01
Sexual abuse		15.7	8.7		6.3	4.2	<0.01
Emotional neglect		7.4	2.5		2.1	0.3	<0.001
Physical neglect		5.9	3.7		3.5	0.9	0.06
HAM-D	20	15.5	8.6	16	0	0	<0.001
YMRS	20	2.1	3.1	16	0	0	<0.01
PANSS above baseline	20	2.6	2.5	16	0	0	<0.001
ZAN-BPD	20	13.7	6.7	16	0	0	<0.001
							
*Medication*	n	*%*					
Antipsychotic medication	12	60					
Antidepressant medication	15	75					
							
*Comorbid diagnoses*	n	*%*					
Total	17	85					
Depression	4	20					
Bipolar affective disorder Ii	4	20					
Eating disorder	3	15					
PTSD	2	10					
OCD	2	10					
Other	2	10					

Abbreviations: BPD, borderline personality disorder; CTQ, childhood trauma questionnaire; HAM-D, Hamilton Rating Scale for Depression; IQ, NART IQ; OCD, obsessive compulsive disorder; PANSS above baseline, PANSS positive symptom score above counting lowest rating as 0 not 1; PTSD, post traumatic stress disorder; ZAN-BPD, Zanarini Borderline Personality Disorder Symptom rating scale; YMRS, Young Mania Rating Scale.
